# Isolation and Characterization of Actinobacteria from Algerian Sahara Soils with Antimicrobial Activities

**DOI:** 10.22088/acadpub.BUMS.6.2.5

**Published:** 2017-07-11

**Authors:** Harir Mohamed, Bellahcene Miloud, Fortas Zohra, José María García-Arenzana, Antonio Veloso, Susana Rodríguez-Couto

**Affiliations:** 1 *Biology of Microorganisms and Biotechnology Laboratory, University of Oran 1 Ahmed Ben Bella, Oran, Algeria.*; 2 *Faculty of Sciences, Natural and Life Sciences Department, Mohamed Boudiaf University, M’sila, Algeria.*; 3 *Institut of Sciences, Faculty of Sciences, Natural and Life Sciences Department, University Center of Ain Temouchent, Temouchent, Algeria.*; 4 *Microbiología* *, Hospital Universitario Donostia, Donostia-San Sebastián, Spain.*; 5 *POLYMAT, University of the Basque Country UPV/EHU, Donostia-San Sebastian, Spain.*; 6 *Ceit-IK4, Water & Health Division, Donostia-San Sebastian, Spain.*; 7 *IKERBASQUE, Basque Foundation for Science, Bilbao, Spain.*

**Keywords:** Actinobacteria, antimicrobial activities, MALDI-TOF MS, Sahara soils, strain identification

## Abstract

Extreme ecosystems can be a source of untapped microorganisms to produce novel bioactive compounds of industrial interest. Consequently, in this work, 32 actinomycetes were isolated from 6 soil samples collected from Algerian Sahara in searching for untapped producers of novel antimicrobial compounds. All the isolates were further subjected to antimicrobial screening against pathogenic bacteria, yeast and fungi. The obtained results indicated that three of the isolates (named C, MS1 and 10) showed antimicrobial activities against most of the tested pathogenic microorganisms. Therefore, these three promising isolates, previously identified as *Streptomyces* by morphological, biochemical and physiological methods, were selected for their subsequent identification by the whole cell matrix assisted laser desorption ionization time of flight mass spectrometry (MALDI-TOF MS) analysis. Thus, the isolates C, MS1 and 10 were identified as *Streptomyces violaceoruber* B263 UFL, *Streptomyces albus* B262 UFL and *Streptomyces badius* B192 UFL, respectively. These results pointed out actinomycetes from Sahara soils as potential sources of novel antimicrobial compounds. Also, MALDI-TOF MS showed to be a robust technique for bacteria identification.

Actinomycetes are ubiquitous Gram-positive aerobic bacteria which present a wide variety of morphologies and some of them, such as those belonging to the *Streptomyces* genus, resemble the filamentous fungi (1). The biotechnological interest of these microorganisms resides in their ability to produce different bioactive compounds. In fact, about two-thirds of natural antibiotics have been isolated from actinomycetes (2,3).

To find novel bioactive compound producers, exploration of ecosystems exposed to extreme environmental conditions is an interesting approach. Hence, research in later years is oriented towards the screening and isolation of actinomycetes from untapped habitats (4). The exploration of such habitats could even provide new taxa which, in turn, could be promising sources of novel bioactive compounds (5-8). In this sense, Algerian Sahara soils, exposed to hard climate conditions, represent particular ecosystems worthy of being explored. In addition, the Algerian Sahara soils have a significant biodiversity (9). Accordingly, the isolation of different actinomycetes strains from Algerian Sahara soils, their antimicrobial activity against pathogenic microorganisms and their characterization via conventional and molecular methods (i.e. MALDI-TOF MS) was investigated.

## Materials and methods

**Sampling**

Six soils samples were collected from different Sahara areas in the south of Algeria, about 15 cm below the surface of the soil. All the soil samples were collected randomly, then packed in zipper bags and stored in a refrigerated container (4.C) during transportation to the laboratory.

The samples were air dried and heated aseptically to remove the undesired Gram-negative bacteria. Appropriate selective media such as yeast extract– malt extract agar medium (ISP2) and peptone yeast extract– malt extract agar medium (GLM) supplemented with actidione (5 µg/mL) and rifampicin (5 µg/mL) were used to promote actinomycetes conditions of growth and prevent fungal contamination (10).

**Isolation and maintenance of actinomycetes**

Ten-fold serial dilutions of soil samples were done using sterile distilled water. The soil suspensions were plated using ISP2 medium supplemented with 40 mg/mL of actidione to inhibit the development of eukaryotic microorganisms. The plates were incubated at 30 C for 7-10 days. Pure colonies were selected by observing the fine filaments around the actinomycete colonies under light microscopy and taken using a sterile inoculation loop. The isolated colonies were maintained on ISP2 agar slants at 4C for subsequent studies.

**Pathogenic bacteria and yeasts**

The pathogenic bacterial strains *Bacillus cereus *ATCC 10876,* Micrococcus luteus *ATCC 533*, Enterococcus faecalis *ATCC 29212*, Lococcus aureus *ATCC 43300*, Staphylococcus aureus *ATCC 25923, *Staphylococcus epidermidis *ATCC 1222*, Escherichia coli *ATCC 25922*, Klebsiella pneumonia *ATCC 43816*, Pseudomonas aeruginosa *ATCC 82827 and *Salmonella enterica *ATCC 14028 and the yeasts *Candida albicans *ATCC 10231 and *Saccharomyces cerevisiae *ATCC 4226 were obtained from the Pasteur Institute in Alger (Algeria). The strains were maintained on Petri plates containing nutrient agar at 4 C and subcultured every 2 months.

**Pathogenic filamentous fungi**

The pathogenic fungi *Fusarium culmorum, Verticillium dahliae *and *Fusarium oxysporum f. sp. albedinis *were obtained from the Institute of Phytopathology and Plant Protection of Messerghine in Oran (Algeria). The fungi were maintained on Petri plates containing potato dextrose agar (PDA) at 4 C and subcultured every 2 months.

**Screening of the actinomycete isolates for antimicrobial activity**

**Fig. 1:**
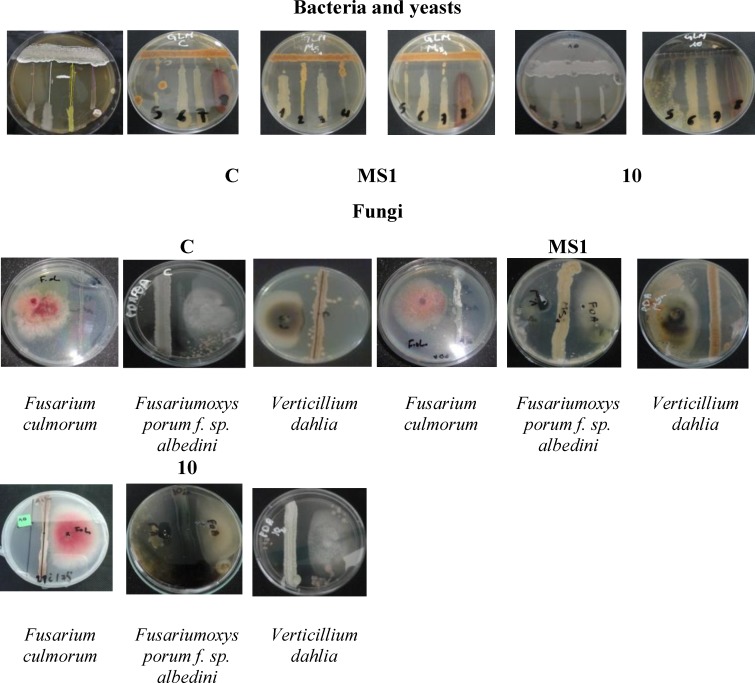
Cross streak method of the isolates C, MS1 and 10 against different pathogenic bacteria, and fungi.

**Fig. 2:**
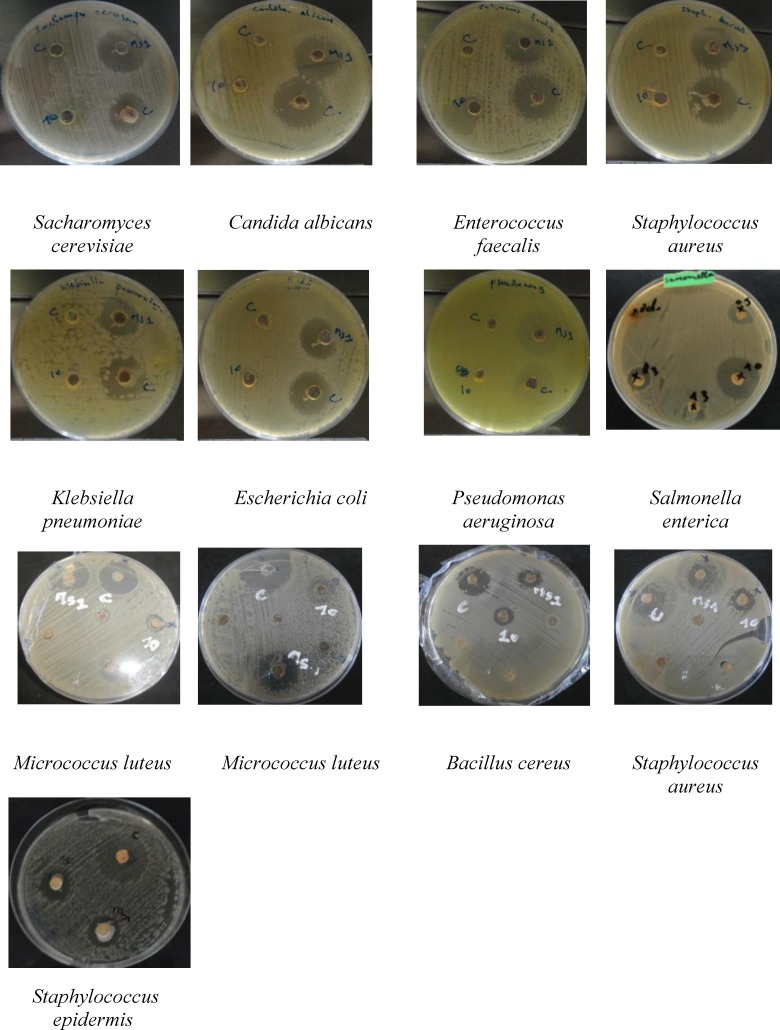
Antimicrobial activity of isolates C, MS1 and 10 against different pathogenic bacteria and yeasts

Primary screening was performed by the cross streak method against selected fungi and yeasts (11) and secondary screening by the agar cylinder method (12) against selected pathogenic bacteria (13). The isolates showing the highest antimicrobial activity were selected for further studies. The antimicrobial activity was evaluated by:

the cross streak method, measuring the distance of inhibition between the pathogenic bacteria and yeasts after incubation at 30 C for 24 h (Figure 1) and fungi after incubation at 25 C for 48 h (Figure 1).

the agar cylinder method, measuring the inhibition zones around the colony of each isolate after incubation at 30 C for 24 h (bacteria and yeast) and 25 C for 48 h (fungi) (Figure 2).

Percentage of inhibition was calculated using the following formula (14).

(%)inhibition=(Rcontrol-Rtest)/Rcontrol x100

where Rtest is the colony diameter of the pathogenic fungus with actinomycete isolates on PDA plates and Rcontrol is the colony diameter of the pathogenic fungus on PDA plates.

The degree of antimicrobial activity of the isolates is classified depending on the mean diameter of the inhibition zone of inhibition. In the present case, the diameter of the zone of inhibition was divided as follows: excellent activity (≥18 mm), good activity (12-15 mm), moderate activity (10-12 mm) and weak activity (≤9 mm). Triplicate samples were performed.

**Characterization of the actinomycete isolates**

The isolates showing antimicrobial activity were characterized morphologically, biochemically and physiologically following the methods given in the International *Streptomyces* Project (ISP) (15).

Color determination was carried out using ISCC-NBS colour charts (16). The micromorphology of the strains was observed by light microscopy after incubation at 30 C for 2 weeks on petri plates containing ISP2 medium. The pigmentation of the aerial mycelium and the structure of sporophores, which are highly charac-teristic and useful in the classification of actinomy-cetes, were observed by cultivating the strains on different ISP media (i.e. yeast extract-malt extract agar (ISP2), oatmeal agar (ISP3), inorganic salts-starch agar (ISP4), glycerol-asparagine agar (ISP5), nutrient agar and Bennett medium). The arrange-ment of spores and sporulating structures was examined microscopically using the cover slip culture method by inserting a sterile cover slip at an angle of 45 C in starch casein agar medium (17,18). A loopful of each isolate was taken from a 7-day old culture, inoculated at the insertion place of the cover slip and incubated at 30 C for 7 days. The cover slip was carefully removed using a sterile forceps and placed upwards on a clean glass slide. The bacterial growth on the cover slip was fixed with a few drops of absolute methanol for 15 min, washed with tap water and then flooded with crystal violet reagent for 1 min followed by washing and blot drying. Finally, the cover slip was examined under microscope using oil immersion lens (100X). Biochemical and physiological characterization of the isolates were performed by streaking them on starch agar plates and incubating them at 30 C for 7 days. After incubation, iodine solution was poured onto the agar and examined for hydrolysis of starch by the production of a clear zone around the microbial growth. Furthermore, the isolates were streaked on gelatine agar plates and incubated at 30 C for 7 days to test for gelatine hydrolysis. After incubation, the plates were flooded with 1 mL of mercuric chloride solution and the diameters of the hydrolyzed zones around the colonies were measured. Also, the isolates were streaked on plates containing skimmed milk agar medium, incubated at 30 C for 7 days and the diameters of the hydrolyzed zones around the colonies were measured. To test sodium chloride resistance, starch casein agar was prepared in three batches and supplemented with 5%, 7% and 10% (w/v) sodium chloride. The medium was autoclaved, poured onto Petri plates and allowed for solidification. Then, the plates were streaked with the isolates and incubated at 30 C for 7 days. Visual observations were done to record the growth of the isolates. Finally, the isolates were streaked on starch casein agar plates and incubated at 25 C,30 C, 35 C, and 40 C for 7 days. The optimum temperature for maximum growth was determined through visual examination (19).

Pathogenic bacteria tested were 1: Bacillus cereus; 2: Micrococcus luteus; 3: Enterococcus faecalis; 4. Staphylococcus aureus ATCC 44300; 5: Staphylococcus aureus ATCC 25923; 6: Staphylococcus epidermidis; 7: Escherichia coli; 8: Klebsiella pneumonia). Pathogenic fungi were: F. c.: Fusarium culmorum; F. o. a.: Fusarium oxysporum f. sp. albedini; V. d: Verticillium dahliae.

**Fig. 3:**
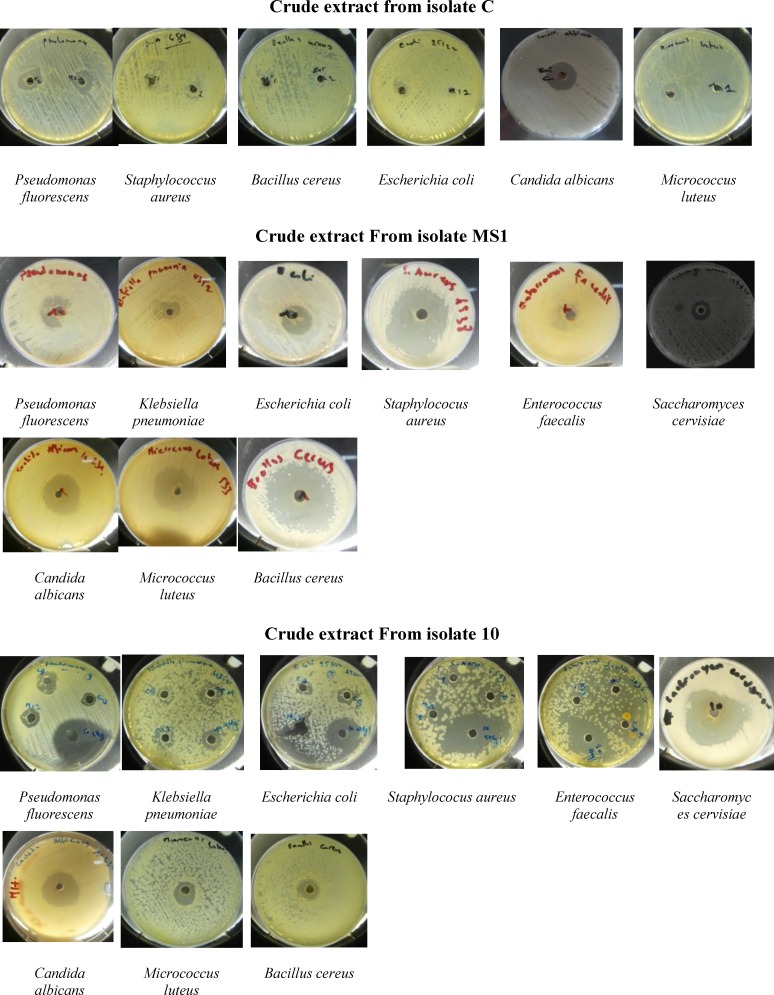
Antimicrobial activity of the crude extract of the isolates C, MS1 and 10 against different pathogenic bacteria and yeasts.

**Table 1:** Antimicrobial activity of strains C, MS1 and 10

**Test organisms**	**Inhibition zone (mm)**		
**Gram-positive bacteria**	**C**	**MS1**	**10**
1. *Bacillus cereus* 2. *Micrococcus luteus* 3. *Enterococcus faecalis* 4. *Staphylococcus aureus* ATCC 44300 5. *Staphylococcus aureus* ATCC 25923 6 .*Staphylococcus epidermidis*	21 28 20 20 21 22	14 35 20 19 22 18	15 22 13 21 20 13
**Gram-negative bacteria**			
7. *Escherichia coli* 8. *Klebsiella pneumoniae* 9. *Pseudomonas aeruginosa* 10. *Salmonella enteric*	19 20 18 11	19 20 18 15	22 22 20 21
**Yeasts**			
11. *Candida albicans* 12. *Saccharomyces cerevisiae*	18 17	20 20	16 11
**Filamentous fungi **(% of growth inhibition)			
13. *Fusarium culmorum* 14. *Verticillium dahliae* 15. *Fusarium oxysporum f. sp. albedinis*	36 34 39	38 35 35	32 40 37

* The values are the mean of triplicate samples with a standard deviation less than 10%

**MALDI- TOF MS identification of the actino-mycetes isolates**

The identification of the actinomycete isolates C, MS1 and 10 by MALDI-TOF MS was perform-ed on a Bruker Microflex system (Bruker, Germa-ny) instrument equipped with a nitrogen laser with an output wavelength of 337 nm used at a repetition rate of 60 Hz. All spectra were acquired in the linear positive mode within a range of 2-20 kDa.

A rapid, on-plate method was used for sample preparation. This method requires a small amount of bacteria which was picked up with a sterile toothpick from the bacteria colony and hand spotted onto a 96-spot polished stainless steel MALDI target plate. The spots were allowed to dry at room temperature and overlaid with 1 µL of MALDI matrix α-cyano-4-hydroxycinnamic acid (CHCA). CHCA was dissolved in a solvent (acetonitrile 50%, water 47.5% and trifluoroacetic acid 2.5%) to a final concentration of 2.5 mg/mL. When the matrix was air dried, the MALDI sample plate was inserted into the spectrometer and spectra were acquired under high vacuum conditions.

MALDI-Biotyper 3.1 software, library version V4.0.0.1 (5.627 MSPs) (Bruker Daltonik GmbH, Bremen, Germany) was used for the identification of each bacteria. This software allows discovering bacteria’s identity by its own unique molecular composition, revealing a characteristic peak pattern, even for reliable differentiation of species: the individual fingerprint. The molecular fingerprint is used for pattern matching. Sophisticated recalibra-tion and statistical algorithms allow robust and accurate identification. Matching scores supported by color codes are used for ranking the results. MALDI Biotyper integrates a ready to-use reference library comprising thousands of individual species and is growing continuously. The identification of unknown bacteria was performed by comparing their spectral fingerprints with those existing in the database (composed for 5627 entries). A matching score based on identified masses and their intensity correlation was generated and used for ranking the results.

Biotyper 3.1 software (Bruker), returned the top 10 identification matches along with confidence scores ranging from 0.0 to 3.0. Estimated values of 2.3 or higher were considered high-confidence scores and indicate that of genus and species identifications is reliable (secure species), score values between 2.0 and 2.29 show that the genus is reliable and the species is probable. Score values between 1.7 and 1.99 were considered intermediate confidence and indicate that the identification of genus was probable. Score values lower than 1.7 were considered “not reliable” evincing that spectra acquisition was insufficient or no peak protein was detected, and further analysis is required for this sample.

**Production, extraction, and detection of antimic-robial compounds**

Each isolate of actinomycetes was cultivated in 500-mL Erlenmeyer flasks containing 100 mL of ISP2 medium (1% malt extract, 0.4% yeast extract, 0.4% glucose, pH 7.2). The flasks were incubated at 30 C for 5 days on an orbital shaker (250 rpm). After that, the culture broth was centrifuged for 20 min at 8000*g* to remove the mycelium. The supernatant was divided into 4 equal volumes (60 mL each) and extracted with 60 mL of an organic solvent. Four different organic solvents ranging from non-polar to polar ones were screened for effectiveness, including n-hexane, dichloromethane, n-butanol and ethyl acetate. The organic phases of strains C and MS1 and the aqueous phase of strain 10 were evaporated to dryness using a Rotavapor (Laborota 4000). To select the best extraction solvents, according to their quantity and antimicrobial activity, the activities of each crude extract of the selected isolates (i.e. C, MS1 and 10) were examined. Briefly, each crude extract was defatted with 1 mL of methanol and subjected to biological assay (disc of 6 mm in diameter, Pasteur Institute) against *Micrococcus luteus* (60 µL per disc) (data not shown). The solvents which gave the highest inhibition diameter were then used for the extraction of the active substances (20).

**Table 2:** Culture characteristics of strains C, MS1 and 10 on different media

**Medium**	**Growth**		**Spore colour**				**Vegetative mycelium**			**Soluble pigment**		
	C	MS1	10	C	MS1	10	C	MS1	10	C	MS1	10
ISP2	+++	+++	+++	Grey	Grey	Yellowish grey	Pale yellow	Blue violet	Brilliant orange yellow	-	-	+Dark green
ISP3	+	+	+	Grey white	Grey white	Yellowish Gray	Pale yellow	Blue violet	Brilliant orange yellow	-	-	-
ISP4	++	++	++	White	White	Yellowish Gray	Pale yellow	Blue violet	Brilliant orange yellow	-	-	-
ISP5	+	+	+	Grey	Grey	Yellowish Gray	Pale yellow	Blue violet	Brilliant orange yellow	-	-	-
Nutrient agar	+	+	+	Grey white	Grey white	Yellowish Gray	Pale yellow	Blue violet	Brilliant orange yellow	-	-	-
Benett medium	+++	+++	+++	Grey white	Grey white	Yellowish Gray	Pale yellow	Blue violet	Brilliant orange yellow	-	-	+Dark green

ISP: International *Streptomyces* Project; ISP2: yeast extract—malt extract agar medium; ISP3: oatmeal agar medium; ISP4: inorganic salts—starch agar medium; ISP5: glycerol—asparagine agar medium.

To test the antimicrobial activities of each crude extract, the agar well-diffusion method on Muller Hinton medium (MHA) was performed. For this, a volume of 25 µL of the crude extract of each strain (i.e. MS1, C and 10) was carefully dispended into each well, allowed to diffuse for 2 h at 4 C and incubated at 37 C for 24 h. 

After incubation, the zone of inhibition (in mm) around each well was recorded.

## Results

**Actinomycetes isolation**

Among the 32 actinomycetes isolated from Sahara soils, 13 isolates showed antibacterial activities against at least one of the pathogenic bacteria by the cross streak method as primary screening and the agar cylinder method as secondary screening. The results revealed that isolates C, MS1 and 10 exhibited broad spectrum activities against pathogenic bacteria (Gram-positive and Gram-negative), especially against *M. luteus, S. aureus *and* S. epidermidis*. Anti-yeast activity was also recorded against *S. cerevisiae *and *C. albicans, *whereas the antifungal activity was moderate (Table 1).

***Morphological, biochemical and physiological characterization of the isolates***

Colonies of strains C, MS1 and 10 grew well on most of the organic media used and were convex and smooth. The aerial mycelium was grey for strains C and MS1 and yellowish grey for strain 10. The substrate mycelium was pale yellow for strain MS1, blue violet for strain C and brilliant orange yellow for strain 10 (http://people.csail.mit.edu/ jaffer/Color/Dictionaries). Abundant dark green diffusible pigments were formed only on ISP2 and Bennett media for the strain 10. The cultural characteristics of the isolates are given in details in Table 2.

The isolates were able to hydrolyze a great number of compounds such as casein, arabinose, fructose, galactose, glucose, mannitol and xylose. They were resistant to sodium azide (0.05 g/L), crystal violet (0.05 mg/mL) and several antibiotics such as ampicillin (20 mg/L), kanamycin (25 mg/L) and tetracycline (30 mg/L). The optimum growth temperature of most isolates was between 25 and 30 C, growth being inhibited at temperatures above 40 C (Table 3).

**Table 3:** Physiological and biochemical properties of strains C, MS1 and 10

	**Isolates**		
**Property**	**C**	**MS1**	**10**
Melanin formation (ISP6 and ISP7) Starch hydrolysis (tryptone soya agar medium) Casein hydrolysis (casein agar medium) Urease production (nitrate peptone broth medium) Gelatine hydrolysis (nutrient gelatine medium) Soluble pigment production (ISP media) H_2_S production (triple sugar iron agar medium) pH range of growth (ISP4) 6 - 9 Temperature range of growth (ISP4) 25-45 C Antibiotic resistance Ampicillin (20 mg/L) Kanamycin (25 mg/L) Tetracycline (30 mg/L) Chloramphenicol (25 mg/L) Chlortetracycline hydroxychloride (30 mg/L) NaCl tolerance NaCl (5% (ISP4) NaCl 7% (ISP4) NaCl 10% (ISP4) Sporophore morphology Straight Spiral Flexous Retinaculum apertum Growth on inhibitory compounds Phenol 0.1% (ISP4) Lysozyme 0.005% (ISP4) Sodium azide 0.01% (ISP4) Crystal violet 0.05% (ISP4)	- + + + + + + - - + + + - R S R R S - + + + - - - + + - + - - - - -	+ + + + + + + - - + + + - R S R S S - + + + - - - + + - - - - - - -	+ + + + + + + + - + + Green + + + + - R S R S S - + + + - - - + + - - - - - ND -

ISP: International *Streptomyces* Project; ISP4: inorganic salts-starch agar medium; ISP6: peptone-yeast extract-iron agar medium; ISP7: tyrosine agar medium. +: Growth; -: no growth; ND: not determined. R: Resistant, S: Sensible.

**Table 4:** Zone of inhibition (mm) of crude extracts produced by strains C, MS1 and 10 on ISP2 (International *Streptomyces* Project yeast extract—malt extract agar) medium using the well diffusion method.

**Test organisms**	**Inhibition zone (mm)**		
**Gram-positive bacteria**	**Strain C**	**Strain MS1**	**Strain 10**
*Bacillus cereus* *Micrococcus luteus* *Enterococcus faecalis* *Staphylococcus aureus* *Staphylococcus epidermidis*	14 15 13 14 12	11 18 --- 12 ---	--- --- 30 35 35
**Gram-negative bacteria**			
*Pseudomonas fluorescens* *Escherichia coli* *Klebsiella pneumoniae*	12 14 15	15 10 ---	36 34 14
**Yeasts**			
*Candida albicans* *Saccharomyces cerevisiae*	17 13	12 ---	31 12

* The values are the mean of triplicate samples with a standard deviation less than 10%.

† ---: no inhibition

***Identification of the isolates by MALDI-TOF MS analysis***

The three isolates C, MS1 and 10 were identified by MALDI-TOF MS as *Streptomyces* *violaceoruber* (NCBI code 1935, score 1.912), *Streptomyces albus* (NCBI code 1888; score 1.261) and *Streptomycete* *badius* (NCBI code 1941; score 1.514), respectively. The first one presented high score and the third one was acceptable. Nevertheless, the second one did not present a high score by MALDI-TOF MS analysis, but with the additional information given by the morphological, physiological, biochemical and cultural characteristics tests, we concluded that it was very likely that strain.

## Discussion

The increased emergence of multidrug resistant organisms makes the treatment of numerous infectious diseases difficult. Hence, the development of novel effective drugs against the abovementioned organisms is needed. For this, the exploration of untapped and extreme habitats can lead to the isolation of novel microorganisms to produce novel bioactive compounds, recently several researchers have shown the potential of extreme habitats as reservoirs of promising antimicrobial compounds producers (4,7,21, 22-26).

Taking into account the results exposed above, the isolation of microorganisms with promising antimicrobial activities from Algerian Sahara soils as a model of an extreme ecosystem was pursued. Among the 32 actinomycetes isolated from Algerian Sahara soils, 3 of them (named as C, MS1 and 10) exhibited broad spectrum antimicrobial activities against different pathogenic bacteria, yeasts and even fungi. These C, MS1 and 10 isolates were identified by combining the results obtained via conventional and molecular methods, as *Streptomyces* *violaceoruber*, *Streptomyces albus* and *Streptomycete* *badius*, respectively. This is not surprising since *Streptomyces* is the most common genus in the actinomycetes order (27). Our results are in agreement with those reported by Kumar et al. (28). As a consquence, they found that *Streptomyces* was the predominant genus in the *actinomycete* strains isolated from soil samples of the Uttarakhand state (India).

The isolated strains (i.e. C, MS1 and 10) are potential producers of bioactive compounds as shown in the antimicrobial activities of their crude extracts against different pathogenic microorganisms (Table 4; Figure 3). These results are in agreement with those reported by different researchers. As a result, Arumugam et al. (24) found that the most common soil bacteria actinobacteria isolated from soil samples of a mangrove forest in India exhibited anti-microbial activity and antibiotic production. Moreover, Elbendary et al. (29) reported antimicrobial activity form actinobateria isolated from farm soils in Egypt. Besidess, *S. albus* G was reported to produce an antibiotic compound (30), *S. violoaceoruber* VLK-4 , isolated from soil samples in India, was shown to produces antimicrobial compound (31) and a bioactive compound was isolated from the culture broth of a *S. badius* strain isolated from Egyptian soil (32).

Strain 10 exhibited an antimicrobial activity higher than strains C and MS1, especially against* P.* *fluorescens *(36 mm) ), *S. aureus* (35 mm), *S. epidermidis* (35 mm), *E. coli* (34 mm) and *C. albicans *(31 mm) (Table 4; Figure 3). These differences can be attributed to their different chemical structures, disintegration during the extraction process (21) and environmental factors (temperature and pH of the crude extract).

It is worthy to mention that the crude extracts from the isolates showed antimicrobial activity against Gram-negative bacteria since, in general, they are more resistant to antimicrobial compounds than the Gram-positive bacteria (29). Lee et al. (33) also reported inhibitory activity against Gram-negative bacteria for actinomycetes isolated from soil samples of the Tanjung Lumpur mangrove forest in Malaysia. On the contrary, Rabia-Boukhalfa et al. (25) detected no activity against Gram-negative bacteria by a halotolerant actino-bacterium, belonging to the genus *Nocardiopis*, isolated from a salt lake soil sample in the Algerian Sahara.

The obtained results point out actinomycetes from Algerian Sahara soils as potential sources of novel antimicrobial compounds. Future research will be required to identify the produced antimicrobial compounds which will involve their purification and the use of different chemical analysis such as HPLC-MS, FTIR and NMR techniques. On the other hand, MALDI-TOF MS has shown to be a fast, reliable and highly robust technique for bacteria identification.
